# ”It Took Me 61 Years to Find What I Really Love”: Older Adult Volunteers’ Engagement in Schools

**DOI:** 10.1093/geroni/igaf122.1747

**Published:** 2025-12-31

**Authors:** Youjung Lee, Laura Bronstein, Francie Keefe

**Affiliations:** Binghamton University, Binghamton, New York, United States; Binghamton University, Binghamton, New York, United States; Broome County Office for Aging, Binghamton, New York, United States

## Abstract

Older adult volunteers in schools benefit multiple generations, including older adults themselves. Given multiple positive impacts of classroom volunteering with youth in one’s later years, it is important to explore older adult volunteers’ experiences in schools. Using a phenomenological qualitative approach, 30 in-depth interviews with older adult classroom volunteers in New York State were conducted (20 in 2019 and 10 in 2024). The interviewees were recruited from the Foster Grandparent Program in the AmeriCorps Seniors, where older adults provide support and mentorship to children. The 2024 interviews included additional questions about volunteers’ experiences related to the pandemic. Interviewees included three African Americans, one Asian, and 16 White older adults (mean age: 63). Two volunteers were male. The qualitative data analyzed using a thematic analysis revealed: (1) Intergenerational engagement benefits youth and their teachers; (2) Older adult volunteers’ benefit from their social engagement in schools; and (3) The volunteers’ proposal for enhancing the Foster Grandparent Program. Findings from this study disclose that the older adult classroom volunteering offers multi-layered engagement: intergenerational engagement for children, older adults, and school professionals, as well as social engagement for older adults from having a new role and identity in later years. The older adult volunteers’ desire for social engagement were exemplified by their eagerness to return to schools during the pandemic. In an aging society, schools can play an innovative role in supporting multiple generations, including older adults, which can be understood from a social capital building perspective.

